# Genotypic and phenotypic variation among *Staphylococcus saprophyticus *from human and animal isolates

**DOI:** 10.1186/1756-0500-3-163

**Published:** 2010-06-10

**Authors:** Britta Kleine, Sören Gatermann, Türkan Sakinc

**Affiliations:** 1 Institut für Hygiene und Mikrobiologie, Abteilung für Medizinische Mikrobiologie, Ruhr-Universität Bochum, D-44780 Bochum, Germany

## Abstract

**Background:**

The main aim of this study was to examine the genotypic and phenotypic diversity of *Staphylococcus saprophyticus *isolates from human and animal origin.

**Findings:**

In total, 236 clinical isolates and 15 animal isolates of *S. saprophyticus *were characterized in respect of the occurrence of 9 potential virulence genes and four surface properties. All strains were PCR positive for the regulatory genes *agr*, *sar* >it>A and *rot *as well as for the surface proteins UafA and Aas. Nearly 90% of the clinical isolates were found to possess the gene for the surface-associated lipase Ssp and 10% for the collagen binding MSCRAMM SdrI. All animal isolates were negative for*sdrI*. Lipolytic activity could be detected in 66% of the clinical and 46% of the animal isolates. Adherence to collagen type I was shown of 20% of the clinical strains and 6% of the strains of animal origin. Most *S. saprophyticus *strains showed hydrophobic properties and only few could agglutinate sheep erythrocytes.

**Conclusions:**

We described a broad analysis of animal and human *S. saprophyticus *isolates regarding virulence genes and phenotypic properties such as lipase activity, hydrophobicity, and adherence. While *S. saprophyticus *strains from animal sources have prerequisites for colonization of the urinary tract like the D-serine-deaminase, out findings suggested that they need to acquire new genes e.g. MSCRAMMS for adherence like sdrI and to modulate their existing properties e.g. increasing the lipase activity or reducing hydrophobicity. These apparently important new genes or properties for virulence have to be further analyzed.

## Introduction

Many genes and characteristics were investigated for the staphylococcal species *S. aureus *and *S. epidermidis*, and the distribution of potential virulence factors among infectious isolates have been studied extensively. In contrast, such study does not exist for the pathogenic *S. saprophyticus*, which is an important cause of urinary tract infections especially in young women [1]. Previously it has been shown that this bacterium is a contaminant of food of animal origin [2]. It was found in 7.1% of rectal swabs from cattle carcasses and 7.3% of rectal swabs from slaughtered pigs.

The urease was the first virulence factor characterized [3] and was found in all *S. saprophyticus *strains. Only four surface proteins have been characterized so far: the collagen-binding serine-aspartat-repeat protein SdrI [4], the uro-adherence factor UafA [5], the fibronectin-binding autolysin Aas [6] and the surface-associated lipase Ssp [7]. *S. saprophyticus *binds different extracellular matrix proteins like collagen [8,4], fibronectin [9,10] and laminin [8] and exhibits different surface properties like hydrophobicity and hemagglutination [11]. According to other staphylococci, an agr- like system was identified [12].

At present, virtually nothing has been reported about the occurrence of all these putative virulence factors among *S. saprophyticus*. Here 236 clinical isolates of *S. saprophyticus *and 15 isolates of animal origin were characterized regarding the existence of these genes and surface properties.

## Methods

### Bacterial strains

A total of 236 *S. saprophyticus *isolates from patients with clinically relevant symptoms and 15 isolates from different animal sources were used in this study. The species were verified using biochemical techniques [13] and equivocal results were resolved by sequencing of the *sodA *gene [14]. The type strain *S. saprophyticus *ATCC 15305 and the already characterized clinical isolate 7108 were used as controls.

### Hemagglutination

For the hemagglutination assay the method described by Gatermann et al. [15] was used with slight modifications. In brief, bacteria were grown in 10 ml peptone yeast extract broth (PY) for 16 h (130 *rpm*, 37°C), cells were harvested (2000 *g*,10 min), washed twice with PBS (140 mM NaCl, 8 mM Na _2_HPO _4_, 2 mM KH _2_PO _4_, pH 7.4) and adjusted at an optical density of 6.0 at 600 nm. The erythrocyte suspension (25 μl, 1 v/v% in PBS) was added to the same volume of serial dilutions of bacteria. Results were read after 2 h incubation at room temperature.

### Lipase activity assay

Lipolytic activity was determined by an agar plate assay containing tributyrylglycerol as described elsewhere [16]. A cleared halo around the colonies smaller than 1 mm after 48 h was defined as negative, whereas a halo greater than 3 mm was considered as highly lipolytic active.

### Collagen adherence assay

Binding of *S. saprophyticus *to immobilized collagen was done as described before [4] with the modification that we adjusted the bacterial suspension to an optical density of 3.0 instead of 6.0 at 600 nm. Collagen adherence was considered as weak if the optical density after staining of the bound bacteria was above 0.15 and as high above 0.25.

### Hydrophobicity

For testing of hydrophobicity bacteria were cultured for 16 h in PH broth (100 *rpm*, 37°C). Cells were washed twice with PBS (pH 7.2) and adjusted to an optical density of 0.3 in 0.9% NaCl. To 1 ml of this suspension 0.5 ml Xylene was added. After gently mixing for 10 min and an incubation of 15 min for phase separation, the optical density of the lower phase was measured. The hydrophobicity index (HPBI) was calculated as: 1-(OD _final_/OD _initial_) x 100. A HPBI above 10% was regarded as weak hydrophobicity, above 40% as high hydrophobicity.

### PCR analysis

Genomic DNA of the *S. saprophyticus *strains was extracted using the QIAamp DNA Mini Kit (Qiagen) suspending the bacteria in the recommended buffer for gram-positive bacteria and addition of 100 μg lysostaphin at the lysis step. For the amplification of the genes three different PCR-programs were used with an initial denaturation at 94°C for 5 min and final extension at 72°C for 7 min. and 35 cycles. Program 1 (sdrI, dsdA) : 94°C 30 s, 50°C 30 s, 72°C 1 min; program 2 (ssp, uafA): 94°C 30 s, 55°C 30 s, 72°C 2 min; program 3: 94°C 30 s, 50°C 30 s, 72°C 30 s. The following primers were used: *sdr*I fwd-GGATAAAAATAGCACAATCGACGAA/rev-CAAGGCTATATTTAGGTGTT, 1624 bp; *ssp *fwd-AAATTCAGAGAATTAGTAGCC/rev-ATGAAGAGTTACGTTCACAC, 3164 bp; *uafA *fwd-CGCGGATCCCCAACATCAGAAGTATATGG/rev-GCGAAGCTTGTGTCAGAAACTAAACCAGC, 2267 bp; *dsdA *fwd-AACGATTTAGCAACACTT/rev-CTATAAGCAAGATTTACC, 1299 bp; *capD *fwd-CGTTCAAGATAAAGAGCG/rev-TTCACCAGATCTAATGCC, 604 bp; *aas *fwd-CAGGTACCGTTAAAGTAC/rev-GATACAACTAACTTGGCAG, 505 bp; *agr *fwd- AATGCGAACCAAATATGCC/rev-GTGCAATCAATCGATGCG, 702 bp; *sarA *fwd-CTTATATTAGCGAACACG/rev-GTTAGCTTCTTTAATGCG, 236 bp; *rot *fwd-TGTTGAAAGATATCGAGG/rev-AATGGATAATAACTGTACG, 237 bp.

### Statement of ethical approval

All procedures performed on the animals were in strict accordance with the National Institutes of Health Guide for Care and Use of Laboratory Animals and approved by the local Animal Care and Use Committee.

## Results

### Distribution of genes

The staphylococcus regulatory genes *agr*, *sarA *and *rot *were present in all tested *S. saprophyticus *strains independent of their origin as was the gene for the D-serine deaminase (*dsd*A). The capsular gene (*cap*D) from the type strain ATCC 15305 could only be found in three clinical isolates. In all *S. saprophyticus *strains the gene for the major autolysin Aas and the surface-protein uro-adherence-factor A (UafA) were detectable. In all animal isolates as well as in 86.9% of the clinical isolates the presence of lipase gene ssp could be shown. Another surface protein SdrI was present in 10.2% of the clinical isolates but not in any animal isolate. All results are summarized in Table 1.

**Table 1:** Distribution of potential virulence genes among isolates of *S. saprophyticus*

	sdrI	ssp	uafA	dsdA	capsule	aas	agr	sar	rot
ATCC 15305	-	-	+	+	+	+	+	+	+

7108	+	+	+	+	-	+	+	+	+

clinical isolates (*n *= 236)	10.2%	86.9%	100.0%	100.0%	1.3%	100.0%	100.0%	100.0%	100.0%

animal isolates (*n *= 15)	0.0%	100.0%	100.0%	100.0%	0.0%	100.0%	100.0%	100.0%	100.0%

### Lipolytic activity

Sixty-six per cent of the clinical isolates showed lipolytic activity on tributyrin agar plates, 15.9% of them a high activity similar to that of the clinical isolate *S. saprophyticus *7108. In case of the animal isolates 46.7% showed lipolytic activity. Of the clinical isolates containing the gene for the lipase Ssp 59.3% possessed detectable lipolytic activity. The results are shown in Table 2.

**Table 2:** Phenotypic properties of *S. saprophyticus strains*.

	Lipolytic activity				Hydrophobicity			Collagen adherence			
	**Total**	**Weak**	**High**	***ssp* ^+^**	**Total**	**Weak**	**High**	**Total**	**Weak**	**High**	***sdrI* ^+^**

ATCC 15305	-	-	-	-			+	-			-

7108	+	-	+	+			+	+		+	+

clinical isolates (*n *= 236)	66.5%	84.1%	15.9%	89.2%	75%	42.7%	57.3%	19.9%	25.6%	74.4%	14.9%

animal isolates (*n *= 15)	46.7%	100%	0%	100%	100%	20%	80%	6.7%	100%	0%	0%

Lipolytic activity was measured on tributyrin agar plates: weak activity: halo > 1 mm, high activity: halo > 3 mm. For measurement of hydrophobicity the hydrophobicity index (HPBI) was calculated from the distribution of the bacteria between an aqueous and an organic solvent phase: weak hydrophobe: HPBI > 10%, high hydrophobe: HPBI > 40%, Adherence to collagen was determined using immobilized collagen type I. The bound bacteria were stained and the optical density was measured: weak adherence: OD > 0.15, high adherence: OD > 0.25.

### Hydrophobicity

Seventy-five per cent of the clinical isolates were hydrophobic whereas all of the animal isolates showed this property (Table 2.). The animal isolates were generally more hydrophobic. Although SdrI seems to be involved in hydrophobicity in strain 7108 no correlation between hydrophobicity and the presence of the *sdrI *gene could be detected. Only 9% of the hydrophobic clinical isolates were sdrI positive.

### Collagen adherence

Adherence to immobilized collagen could be observed in 19.9% of the clinical isolates and in 6.7% of the animal isolates. The intensity of collagen binding in human isolates was much higher than in animal isolates. 30% of the *sdrI *positive strains and 18% of the *sdrI *negative strains showed binding to collagen (Table 2.).

### Hemagglutination

Hemagglutination of sheep erythrocytes was shown by 15.7% of the clinical isolates and 6.7% of the animal isolates. No correlation with a known protein could be identified because both proteins that are thought to be responsible for hemagglutination, UafA and Aas were found to be present in all tested *S. saprophyticus *isolates.

## Discussion

In the present study 236 *S. saprophyticus *strains isolated from patients and 15 strains of animal origin were characterized for several putative virulence genes by PCR and for phenotypic characteristics often associated with virulence. In all strains analyzed in this study the regulatory genes *agr*, *sar*A and *rot *could be detected even though a regulatory function of these genes in *S. saprophyticus *has not been shown yet. Moreover, all strains were found to possess the gene for the D-serine deaminase (*dsd*A) which is also characteristic for uropathogenic *E. coli*. The high D-serine concentration in urine is toxic to bacteria unless they possess dsdA. Even though *S. saprophyticus *is considered as a natural colonist of the animal skin it seems to have already the basic prerequisite for colonization of the urinal tract.

Lipases are thought to be involved in pathogenesis whether in nutrition [17] or adherence [18,19]. *S. saprophyticus *living on the skin which is low in nutrition but rich in lipids may use their lipase for this purpose. But Ssp is also an important factor during urinary tract infections (data not published) and this may be reflected in the generally higher lipase activity we could observe. Over 60% of the clinical isolates showed lipolytic activity and most of them possessed the lipase gene *ssp*. Because *S. aureus *or *S. epidermidis *have more than one lipase gene [17-20] the lipolytic *ssp*-negative *S. saprophyticus *strains may possess a second lipase. Adherence to extracellular matrix proteins or epithelial cells is a crucial step in bacterial colonization. *S. saprophyticus *binds to different proteins of the extracellular matrix and we could show that in *S. saprophyticus *strain 7108 the surface protein SdrI is responsible for binding to collagen type I [4]. Nearly 20% of the clinical isolates showed adherence to collagen but only about 15% of these strains contained the *sdrI *gene. The second surface protein UafA is not involved in adherence to collagen (data not shown) so a further unknown MSCRAMM can be postulated which is not present in *S. saprophyticus *strains 7108 and the sequenced strain ATTC 15305. In animal isolates collagen binding is less prevalent and less intense and *sdrI *could not be detected in any of these strains. *S. saprophyticus *may have acquired these MSCRAMMs for adapting to their human hosts. Hemagglutination and hydrophobicity are also adherence properties. Most strains characterized are hydrophobic but only very few strains showed hemagglutination despite the fact that all possessed Aas as well as UafA both proteins that are reputedly responsible for hemagglutination [15,5]. Often a capsule is thought to mask such effects of surface proteins but the capsular gene capD of the strain ATCC 15305 could only be detected in 3 strains. One positive strain, *S. saprophyticus *9325, is hemagglutination negative and hydrophilic but degradation of the capsule turned this strain hydrophobic (not shown). At least in this case the capsule seems to mask hydrophobicity. Further capsular genes or regulation mechanisms are possible.

Phenotypic and genotypic characterization of *S. saprophyticus *strains was done for various genes and properties considered to be involved in pathogenesis. The findings suggested that *S. saprophyticus *possesses more surface proteins especially for adherence to extracellular matrix proteins and eukaryotic cells than the four proteins that have been identified.

## Conclusions

We described a broad analysis of animal and human *S. saprophyticus *isolates regarding virulence genes and phenotypic properties such as lipase activity, hydrophobicity, and adherence. While *S. saprophyticus *strains from animal sources have prerequisites for colonization of the urinary tract like the D-serine-deaminase, out findings suggested that they need to acquire new genes e.g. MSCRAMMS for adherence like sdrI and to modulate their existing properties e.g. increasing the lipase activity or reducing hydrophobicity. These apparently important new genes or properties for virulence have to be further analyzed.

## Competing interests

None of the authors of this paper has a financial or personal relationship with other people or organizations that could inappropriately influence or bias the content of the paper.

## Authors' contributions

BK performed the experimental work and wrote the manuscript. ST wrote and edited the manuscript and coordinated the study and SG helped to edit the manuscript. All authors read and approved the final manuscript
